# Poisonous Effects of Soda Water from Copper Fountains and Lead Pipes

**Published:** 1854-07

**Authors:** R. Ogden Doremus


### ARTICLE XIX.

### *Poisonous Effects of Soda Water from Copper Fountains and Lead Pipes.* By R. Ogden Doremus, M. D.

Having, within a few days, had several friends relate their sudden illness after taking a single glass of soda water, and suspecting some poisonous impregnation to be the cause, I was induced to obtain several gallons of this favorite beverage, from different parts of the city, and to submit them to a chemical examination.

The substance which first attracted attention was *copper*.

This was very abundant in soda water obtained from several obscure shops, where it was presumed the traffic was limited, and consequently the acid water remained longer in the copper condensers. It was so evident that, on boiling off the excess of carbonic acid gas, a green scum made its appearance, which, on further evaporation, settled. This was carbonate of copper, previously held in solution by the carbonic acid.

The amount of metallic copper in a quart was one grain and a half!

Soda water obtained from the same establishment on different days, was found to contain varying amounts of poisonous carbonate.

The source of this copper, and the cause of these differences, may be accounted for in several ways.

The copper condensers purport to be tinned internally; but where they have been in use a long time, the tin, by chemical and mechanical action, has been removed, at least in part; thus exposing a surface of copper to the corrosive action of the carbonic acid, aided by sulphuric acid, which is occasionally found in the soda water.

Although the carbonate of copper is insoluble in *pure* water, it is capable of being held in solution in water highly charged with carbonic acid gas; for the soda water which yields this green scum after discharging the gas, is clear and colorless previous to the operation.

The soda water drawn shortly after charging the condenser, would necessarily yield less copper, on analysis, than that obtained from the same fount after having several days to exert its corrosive influence. Again, the tinning (for all are professedly thus lined) would be more perfect in some than in others—dependent not only on the length of time the condensers had been used, but also on the completeness of the original coating. I have been informed that, in order to facilitate the flow of the tin, soft solder is at times resorted to, or the copper is washed with a salt of mercury. Under these circumstances, the chemical and electrical action would be rather complicated, and the soda water possessed of remarkable *medicinal virtues.*

The second poisonous compound which, from its abundance, demanded investigation, was a white precipitate, the carbonate of lead. This was found, to a greater or less amount, in most of the waters examined.

In the quart whence the grain and a half of copper was obtained, 0.65 of a grain of metallic lead was found.

The chief source of this impregnation is the lead pipe used in many fountains to convey the carbonated water from the condensers to the jet.

It is an established fact, that the free carbonic acid found in spring waters is capable of dissolving or façilitating the solution of many of the salts of lead, such as are found encrusting lead pipes which have been used for conducting said waters.

By the investigations of Dr. Ellet, published in this city last year, it was clearly shown that even the trivial amount of carbonic acid found in Croton water is sufficient to act upon the lead pipes.

This lead may be readily found in any kettle which has been used for boiling the Croton water passed through a lead pipe, by adding a little acetic acid to it. The acetate of lead will respond to sulphureted hydrogen, by assuming a black tint (the sulphuret of lead,) or a yellow tint with the iodide of potassium, &c.

Since carbonic acid is possessed of such solvent powers, soda water, which is surcharged with it, must become poisonously contaminated by contact with lead, either in the pipes or the soldering; and as much of the tin of commerce is alloyed with lead, even this metal, to which we look for protection, may be another source of evil.

Many are impressed with the belief that the first few glasses may be impregnated with lead to an injurious extent; and hence the custom, in the most respectable establishments, of discarding the soda water which is first drawn, and has lain in the tube over-night.

Wherever lead pipes are used to conduct the water to the jet, and especially where, in order to secure a cool draught, from thirty to sixty feet of lead pipe are coiled in a tank and covered with ice, the highly acid liquid must necessarily dissolve the metal, and communicate the poison to all contained within the condenser.

These remarks are not applicable to pipes of pure tin, or of lead properly coated with tin.

I have examined the soda water obtained from a manufactory where it is bottled, but could discover neither copper nor lead.

The effervescent liquid which is at times "palmed off" upon the public, made by forcing atmospheric air into water (most truly, "aërated water,") would, from the very want of the carbonic acid, be nearly free from those contaminations.

It might be asked, "if these poisonous bodies exist in soda water, why are not the effects more commonly known?" I would reply, they are more generally known than is supposed.

Since commencing these investigations, I have learned from several medical friends, that a coppery taste, violent vomiting, colic pains, purging, &c., have not been uncommon results from such draughts; and most with whom I have conversed have experienced these effects personally.

In Dr. Mitchell's Therapeutics mention is made that soda water from old copper fountains is strongly marked with the copper taste.

My assistant informs me that five years since, while in a drug store, he observed that vomiting and other symptoms of poisoning by copper followed frequently after drinking soda water, and that many thought it was cholera; and after being similarly affected himself, he tested the water and found copper.

I am informed by a resident of St. Louis, that while the cholera prevailed, most persons abandoned the use of soda water; it was a common remark, "Mr. —— took a glass of soda water, and was immediately attacked with cholera."

Probably the syrups which are the usual accompaniments of the soda draught, act in many cases as an antidote; for although the efficacy of sugar in this respect, as originally proposed by

Duval, was denied by Orfila, it has lately been reasserted by Postel.

I regret that, for want of time, I have not been able to complete other experiments on this subject; yet, as I am convinced that in many cases this poisoned soda water has proved the exciting cause of cholera in those predisposed to this disease, and in others, that it has by its inherent properties been injurious to health or destructive to life; and as at this time the cholera question is again agitating the public mind, I have thought it advisable to relate the results of this partial investigation.

With the knowledge of these facts, we may conclude that although soda water may be retained in a *well-tinned* copper condenser, and discharged through a *thoroughly-tinned* lead pipe, without poisonous impregnation; yet, as any imperfection in the tinning of either, or long or careless usage, may expose the copper or the lead (or both) to the solvent powers of this carbonic acid, and thus render the beverage dangerous, therefore, these vessels should be discarded or only permitted in the hands of trustworthy persons.

Condensers of stone, of iron, or of the purest block tin supported by iron bands, or of gutta percha, aided in a similar manner, would be free from poisonous impregnation. Conducting pipes of these latter materials are likewise unobjectionable.

In another paper I shall present the results of more extended investigations, and shall be indebted to any physicians or pharmaceutists who feel disposed to assist in this work of common interest, by favoring me with reports of cases, or samples of suspected liquid for analysis. If those engaged in the fabrication of this article would afford an opportunity of examining some of the old soda fountains, it might aid materially, and perhaps result in the suggestion of better methods of protection.—*New York Med. Gazette.*

